# Origin of Mg-rich clay minerals in the first member of Maokou Formation in the middle Permian in the Central and Southern Sichuan Basin (China) and their implications on supergene and hypogene fluid regimes

**DOI:** 10.1371/journal.pone.0338303

**Published:** 2025-12-05

**Authors:** Wancang Tan, Xiandong Wang, Pengfei Niu, Xueying Jin, Liang Zhao, Ying Wang, Yaguang Li, Yu Zhang

**Affiliations:** 1 Research Institute of Exploration and Development, Petrochina Daqing Oilfield Company, Daqing, Heilongjiang, China; 2 Chengdu Exploration and Development Research Institute of PetroChina Daqing Oilfield Company, Chengdu, Sichuan, China; Indian Institute of Technology (IIT) - Bombay, INDIA

## Abstract

The first member of Maokou Formation (MF1) in the Sichuan Basin is characterized by marl strata that serve as effective natural gas reservoirs. Notably, the development of MCMs (magnesium-rich clay minerals) plays a significant role in enhancing these reservoirs, especially sepiolite and talc. The diagenesis of sepiolite in the MF1 (Middle Permian) of the central and southern Sichuan Basin was investigated through core and thin section observations, combined with X-ray diffraction (XRD), scanning electron microscopy (SEM), whole-rock major and trace element analysis, and LA-ICP-MS elemental analysis. MCMs occur in various forms, including lamellae, lens-like structures, clastic, strip-like, and metasomatic bioclasts. The MCMs appear gray-black on core samples, with XRD analysis indicating sepiolite and talc as the primary constituents. Under scanning electron microscope, these MCMs are typically observed as granular particles dispersed alongside quartz, while some replace bioclasts in a concentric zonal pattern. Based on rare earth element (REE) characteristics, MCMs can be classified into two genetic categories: sedimentary and hydrothermal types. Sedimentary MCMs exhibit a negative δEu anomaly, high Al/(Al + Fe + Mn) and Y/Ho ratios, and lack heavy REE enrichment. In contrast, hydrothermal MCMs display the opposite characteristics, positive δEu anomaly, low Al/(Al + Fe + Mn) ratio, and elevated concentrations of hydrothermal-related elements. Sedimentary MCMs form through chemical precipitation or metasomatic processes in silicon- and magnesium-rich seawater, while hydrothermal MCMs result from siliceous hydrothermal activity affecting magnesia-rich carbonate rocks. The diagenetic evolution of MCMs contributes to the formation of unconventional reservoirs in MF1 strata by creating organic and clay shrinkage pores. Thus, MCMs-enriched marl represents a promising target for oil and gas exploration within MF1 strata in the Sichuan Basin.

## Introduction

In the global pursuit of enhanced energy security and diversified hydrocarbon sources, the exploration and optimization of unconventional carbonate reservoirs have emerged as a critical area of research [1–3]. These complex geological systems, often characterized by intricate pore structures and significant clay content, present unique challenges and opportunities for efficient resource extraction [3–6]. The Sichuan Basin is a significant oil-bearing region in western China. Recent advancements in oil and gas exploration have revealed industrial gas flows in several wells within the first member of Maokou Formation (MF1), particularly in the Hechuan and Fuling areas located in the basin’s central and eastern regions [7,8]. Petroleum exploration has confirmed that the MF1 Formation is primarily composed of micrite and argillaceous limestone interlayers [9], both of which are rich in bioclasts and exhibit high clay content, particularly in the argillaceous limestone. These characteristics suggest MF1 acts as a self-sourced and self-stored carbonate reservoir [10].

The formation of MF1 marl has been extensively studied, with sedimentary and diagenetic origins widely recognized [11,12]. Differential deposition, influenced by microtopographic variations of shoals and inter-shoal areas, leads to micrite dominance in elevated regions and marl prevalence in lower areas [13]. High magnesium-rich clay mineral content, particularly of sepiolite and talc, is notable in MF1 marl [10]. Sepiolite, a high-magnesium, low-aluminum mineral with a chain-layered structure, is rare and requires specific sedimentary conditions for its formation [14,15]. Despite its significance, the genesis of MCMs in MF1 marl remains controversial. Potential origins include chemical precipitation in silicon- and magnesium-rich seawater under arid to semi-arid climates, transportation of authigenic minerals along basin margins, or alteration from montmorillonite, volcanic sediments, or magnesia-rich carbonate rocks [16–22]. This study investigates the MCMs genesis through core samples obtained from central and southern Sichuan. Employing the SEM, XRD, whole-rock elemental analysis, and laser ablation inductively coupled plasma mass spectrometry (LA-ICP-MS), the research aims to elucidate the mineralogical transformation processes within the MF1 reservoir. Through systematic characterization of the marl reservoir, the study further delineates the key geological controls on reservoir development, providing critical insights for hydrocarbon exploration strategies in Sichuan Basin’s marl reservoirs.

## Geological background

The Sichuan Basin, a diamond-shaped structure, comprises five tectonic units: the North Sichuan low-gentle tectonic belt, West Sichuan low-steep belt, South Sichuan low-steep belt, Central Sichuan gentle belt, and East Sichuan high-steep belt [23]. Although the study area has experienced multiple stages of tectonic movements, the current tectonic setting in central Sichuan is relatively stable, characterized mainly by gentle anticlines and synclines with relatively weak stratigraphic deformation. While some minor fault activity and structural adjustments may still occur, active formation of new folds and faults within the basin appears limited at this stage. In contrast, the southern Sichuan region undergoes intense tectonic deformation. Influenced by the tectonic belts at the basin margins, it has developed numerous folds and faults with diverse tectonic styles. Well D1 is located between Chongqing and Luzhou. From the current Maokou Formation top surface structure, it lies at the high point of the South Sichuan low-steep structural belt. Well H1 is located in the southeastern part of the Central Sichuan gentle belt.

**Fig 1:**
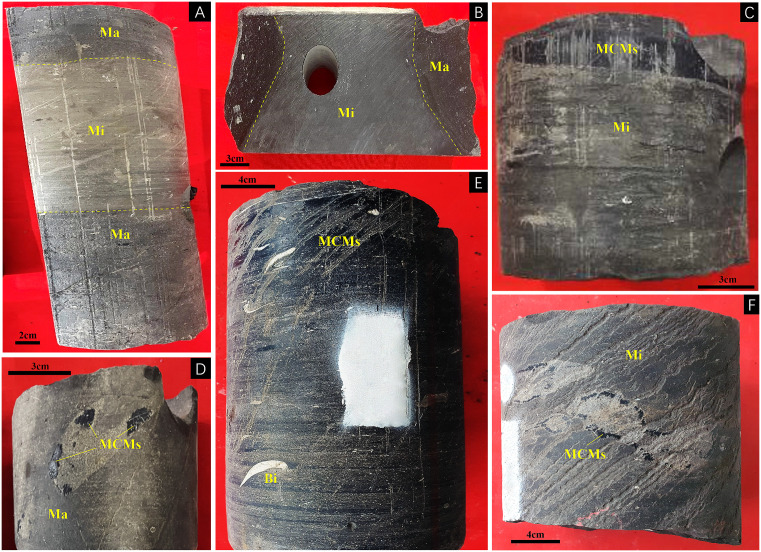
Lithological characteristics of MF1 and MCMs occurrence types in the Central and Southern Sichuan Basin. **A)** Interbedding of micrite and marl, H1 well, 4249.3m; **B)** Interbedding of micrite and marl, H1 well, 4258.4m; **C)** Lenticular MCMs, H1 well, 4263.6m; **D)** MCMs intraclasts, D1 Well, 2723.26m; **E)** MCMs strip, H1 well, 4247.8m; **F)** MCMs metasomatic biological shell, D1 Well, 2679m; Bi = bivalve; Ma = Marl; Mi = micrite.

In both the central and southern Sichuan regions, strata from the Sinian to the Quaternary are well – developed, showing a relatively complete stratigraphic sequence. The Maokou Formation belongs to the Guadalupian Series of the Middle Permian, mainly composed of marine carbonate deposits. Overall, it demonstrates a vertical evolution of the carbonate platform from an open to a restricted environment, accompanied by sea – level fluctuations and changes in the paleogeographic pattern [24]. In the central Sichuan region, the characteristic lithology is intra – platform shoal facies limestone, while in the southern Sichuan region, the dominant facies is platform – margin bio – reef shoal facies [25]. According to lithology, the Maokou Formation is divided into four members [26]. From top to bottom, they are as follows: the Fourth Member consists of interbedded limestone and calcareous mudstone, with a purplish – red ferruginous mudstone weathering crust developed at the top, which reflects tidal flat deposition with shallowing water and exposure events under a regressive background [27]. The Third Member is composed of gray – black thin – to medium – bedded micritic limestone intercalated with siliceous bands and contains pyrite, indicating a weakly reducing environment in a restricted platform [28]. The Second Member is characterized by the development of light – gray thick – bedded massive bioclastic limestone and dolomite, reflecting a high – energy shoal facies [29]. MF1, the lowest and oldest member, primarily consists of rhythmically developed dark gray to gray-black marl and micrite, with abundant bioclasts and visible bitumen-filled fossil cavities (Fig 1A and 1B).

## Materials and methods

In the Central and Southern Sichuan Basin, PetroChina Oilfield Company’s H1 and D1 wells yielded a total of 16 core samples from the MF1 strata. These samples were collected without the need for a permit. Specifically, well D1 contributed 10 samples, while well H1 provided 6. All 16 samples were cut and polished to prepare a thin section for petrographic studies using a polarizing microscope, micro-textural analysis using SEM, and in-situ elemental (major, trace, and REE) analysis using LA-ICP-MS. The same samples were powdered into a 200-mesh (A.S.T.M.) fraction and utilized for whole-rock mineral identification using XRD and for determining major and trace element concentrations by ICP-MS.

### Mineralogical characterization

The analyses, including thin section identification and argon ion polishing-scanning electron microscopy, were conducted at the State Key Laboratory of Petroleum Resources and Exploration, China University of Petroleum (Beijing). The thin section observation was conducted using a polarizing microscope of Nikon E600Pol Type 10, while the scanning electron microscopy was carried out using a Quanta 250FEG field emission environmental scanning electron microscope.

### X-ray xiffraction (XRD) analysis

X-ray diffraction (XRD) was employed to obtain diffraction patterns, which are then analyzed to determine the composition of materials. The XRD analyses were conducted at the China University of Petroleum (Beijing). For each sample, 40 g of 200-mesh powder was ground using a planetary ball mill, then treated through natural drying before whole-rock XRD analysis. The XRD experiments were carried out using a Bruker D2PHASER diffractometer. The diffractometer utilized a Cu-Kα radiation source (λ = 0.154 nm) with a scanning rate of 2°/min and a scanning range of 3° to 65°, operating at 40 kV and 30 mA to record the mineral XRD patterns. After the analysis, RockQuan and ClayQuan software were used to quantify the results and obtain data on the phase composition. Then, approximately 10 g of dried sample was placed in a beaker for the separation of clay minerals, and 100–200 mL of 0.05 mol/L sodium hexametaphosphate solution was added. The mixture was stirred with a magnetic stirrer for 3 hours to ensure complete dispersion of the clay minerals. The suspension was then transferred to a centrifuge tube and centrifuged at 4000 rpm for 10 minutes. The supernatant containing dispersed clay minerals was carefully decanted and transferred to a new container. This centrifugation process was repeated three times to achieve thorough separation of clay minerals from other mineral phases. A 0.8 mL aliquot of the clay suspension was then pipetted onto a glass slide and dried in an oven at 50°C to prepare an oriented clay slide. The slide was saturated with ethylene glycol vapor at 60°C for at least 8 hours, followed by heating at 550°C for a minimum of 2.5 hours. After cooling to room temperature naturally, the prepared slide was ready for XRD analysis.

### Trace element analysis

Whole-rock trace element analysis was performed using an AB104L Axios-mAX wavelength dispersive X-ray fluorescence spectrometer. The whole-rock samples were first crushed to a particle size of less than 0.125 mm, using a jaw crusher for initial coarse crushing and a ball mill for fine grinding. Then, they were dried at 105°C to 110°C for 2 hours and cooled in a desiccator. A 5−10 g of the dried sample was weighed into a platinum crucible, and lithium tetraborate flux was added at a 1:5–1:10 ratio. The mixture was stirred well and heated gradually to 1050°C ± 50°C in a high-temperature furnace for 15−20 minutes until a homogeneous glass bead is formed. Before analysis, the spectrometer was calibrated with certified reference materials including JG-2 (granite) and NIST SRM 610 (glass).

The samples were processed to a particle size of less than 0.125 mm, dried at 105°C to 110°C for 2 hours before analysis, and then cooled to room temperature in a desiccator. The test material was placed in a platinum crucible, gradually heated to 1050°C ± 50°C, and burned to a constant mass. The difference in mass after heating represents the loss on ignition. Trace and rare earth element analysis was conducted using an ELEMENTXR plasma mass spectrometer (ICP-MS). The samples were dissolved using hydrofluoric acid and nitric acid in a closed sample solubilizer. The hydrofluoric acid was evaporated on an electric heating plate, followed by dissolution with nitric acid. After dilution, the samples were analyzed using the ICP-MS external standard method. The analysis was completed at the Beijing Institute of Geology of the Nuclear Industry, with a total of 16 samples analyzed.

### In-situ LA-ICP-MS analysis

The in-situ LA-ICP-MS analysis was conducted at Beijing Createch Testing Technology Co., Ltd. The instrument used was an Analytik Jena ICP-MS system, coupled with a RESOLution 193 nm excimer laser ablation system. A total of 4 slices were delivered for analysis. The laser spot beam used for ablation had a diameter of 50 μm, a frequency of 4 Hz, and an energy density of approximately 5 J/cm2, with high-purity helium as the carrier gas. Before testing, the instrument was calibrated with NIST610 to ensure optimal performance. The LA-ICP-MS laser ablation sampling was carried out using a single-point ablation method. With this approach, sampling points were solely limited to the MCMs. During the test, a blank background was first collected by blocking the laser beam for 20 seconds, followed by continuous ablation of the sample for 45 seconds. After the ablation, the sample injection system was cleaned by purging with helium for 20 seconds. A set of calibration standards (NIST610, NIST612, BHVO-2G, BCR-2G, and BIR-1G) was introduced every 10 ablation points to ensure accurate elemental quantification. Offline data processing (including sample and blank signal selection, instrument sensitivity drift correction, and element content calculation) was performed using ICP-MS Data Cal software.

## Results

### Petrographic observations and quantitative evaluation of minerals

**Fig 2:**
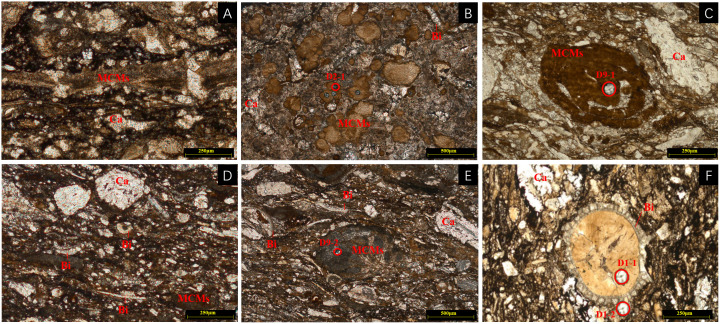
Microscopic characteristics of MF1 MCMs in the D1 well, southern Sichuan Basin. **A.** Banded MCMs, D3, 2681.33 m; **B.** Clumped MCMs, D2 sample, 2680.30m; **C.** Metasomatized MCMs, D9, 2724.60 m; **D.** MCMs metasomatic clasts, D10, 2728.48 m; **E.** MCMs aggregates, D9, 2724.60 m; **F.** MCMs metasomatic clastic body cavity, D1, 2676.66 m; Bi = bivalve; Ca = Calcite; D2-1, D9-1, D9-2, D1-1 and D1-2 are the spots of LA-ICP-MS.

The MCMs are typically gray or black in color, and mainly occurs in three forms in the MF1 core: lenticular (Fig 1C) and intraclasts (Fig 1D) distribution, striped (Fig 1E), and as bioclast infillings or cementing cavities (Fig 1F). Under plane-polarized light, the MCMs appear light brown with plate-like, scaly, and fibrous structures. The mineral forms lamellar or lenticular aggregates and coexists with bioclasts and calcite, often appearing as layered aggregates (Fig 2A). Intraclastic MCMs are dispersed within the limestone matrix, occurring in platy and massive forms embedded in micritic calcite (Fig 2B, 2C, 2D, and 2E). The MCMs are also observed replacing biological fragments, including crinoids, brachiopods, bivalves, foraminifera, ostracods, and algae, filling or re-cementing biological cavities (Fig 2F).

**Fig 3:**
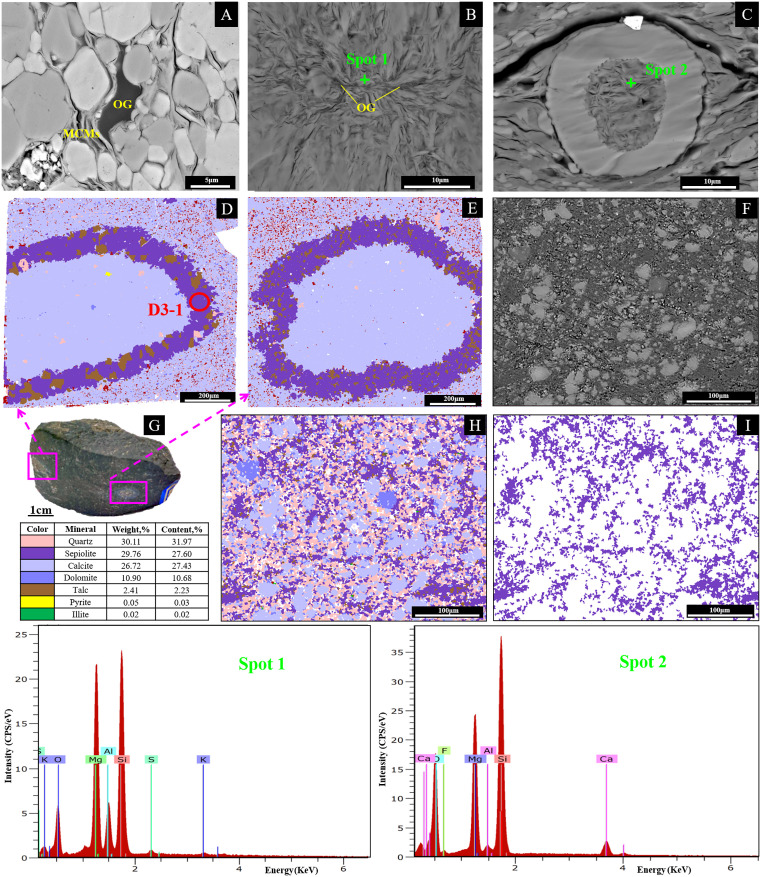
MF1 minerals under scanning electron microscope in D1 and H1 wells. Fig 3A and 3B shows fibrous MCMs from samples H2 and H4. While Fig 3C displays MCMs within biological debris from Sample H2. Both EDS spots (designated locations, spot 1 and spot 2) consistently reveal the compositional characteristics of MCMs. Fig 3D and 3E were different bioclasts from D3 sample in Fig 3G; Figs 3F, 3H, and 3I display the same area from Sample D9. Fig 3H represents the full mineral mapping of Fig 3F, while Fig 3I retains only the sepiolite mineral phase. The color legend in the lower-left corner also includes the mineral composition of Fig 3F. The elemental characteristics of Spot 1 and Spot 2 are shown in S1 Table.

Under scanning electron microscope (SEM), MCMs occur in flaky, scaly, and fibrous forms (Fig 3A, 3B and 3C), distributed in marl as bands of mineral aggregates and diffuse granular particles. Due to its strong adsorption capacity, significant amounts of organic matter are adsorbed in surrounding areas (Fig 3A and 3B). Some MCMs occur in a banded form replacing biological shells (Fig 3D, 3E, and 3G) with some talc and quartz. Inside the shells, sepiolite and talc are rarely observed, except for calcite. Other MCMs are present as granular forms in the pores around calcite and dolomite, accompanied by granular quartz (Fig 3F and 3H). The MCMs exhibit non-uniform distribution, occurring as dispersed particles among calcite, quartz, and other minerals, with localized concentrations reaching 27.6% of the total composition.

### Phase identification and quantification by the XRD experiment

The MF1 minerals in the study area primarily consist of calcite, dolomite, quartz, and clay minerals. The clay minerals include illite, kaolinite, chlorite, talc, sepiolite, and illite/smectite mixed layers (Table 1).

**Table 1:** XRD mineral content of MF1 strata in the D1 and H1 wells.

Sample	Non clay mineral content/%			Clay mineral content/%					
	quartz	calcite	dolomite	illite	kaolinite	chlorite	talc	sepiolite	illite/smectite mixed
**D1**	9.5	75.1	1.7	2.3	0.2	0.5	8.2	0	2.5
**D2**	5.2	38.4	0.5	0.5	0	0	37.3	6.3	11.8
**D3**	2.5	79.7	1.2	0.8	0	0	7.2	1.5	7.1
**D4**	3.2	84.2	1.3	2.8	0.3	0.2	8.0	0	0
**D5**	9.1	47.9	1.1	0	0	0	41.2	0.7	0
**D6**	5.0	50.9	0.5	0	0	0	43.6	0	0
**D7**	3.6	37.1	1.8	0	0	0	57.1	0.4	0
**D8**	8.1	32.7	11	0	0	0	47.2	1.0	0
**D9**	16.7	16.5	7.2	1.3	5.4	5.9	22.2	23.2	1.6
**D10**	0.3	19.6	47.9	0	0	0	32.2	0	0
**H1**	1.3	94	4.7	0	0	0	0	0	0
**H2**	19.1	41.2	10.6	1.7	0.9	0.5	4.5	0	21.5
**H3**	9	91	0	0	0	0	0	0	0
**H4**	5.5	83	2	0	0	0	9.5	0	0
**H5**	5.5	91.3	3.2	0	0	0	0	0	0
**H6**	2.6	97.4	0	0	0	0	0	0	0

**Fig 4:**
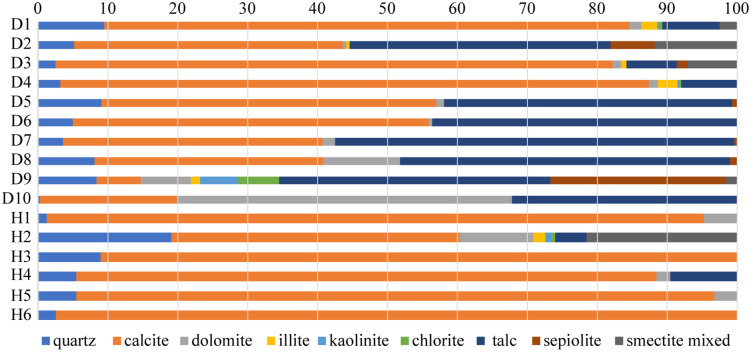
XRD mineral composition of the MF1 strata in the D1 and H1 wells.

In D1 Well, the marl is predominantly composed of calcite, dolomite, quartz, and clay minerals, with sepiolite and talc being the most abundant clay minerals, followed by illite, kaolinite, chlorite, and illite/smectite mixed layers (Fig 4). The calcite content ranges from 16.5% to 84.2% (average 48.2%), dolomite content ranges from 0.5% to 47.9% (average 7.4%), quartz content ranges from 0.3% to 16.7% (average 6.3%), and clay minerals range from 11.3% to 59.6% (average 38.1%). Among the clay minerals in D1 Well, talc content ranges from 7.2% to 57.1% (average 30.4%), sepiolite content ranges from 0% to 23.2% (average 3.3%), illite content ranges from 0% to 2.8% (average 0.8%), and kaolinite content ranges from 0% to 5.4% (average 0.6%). Chlorite content ranges from 0% to 5.9% (average 0.7%), and the illite/smectite mixed layer content ranges from 0% to 11.8% (average 2.3%).

**Fig 5:**
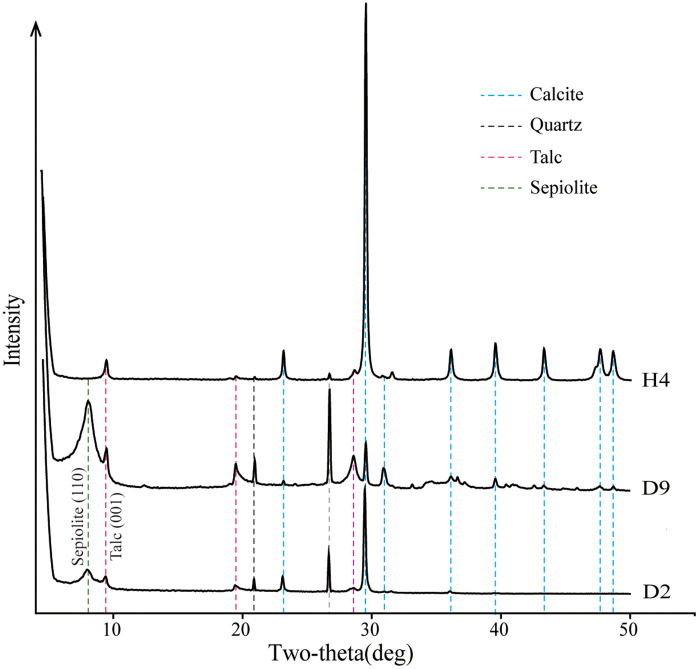
XRD mineral composition of three representative MF1 whole-rock samples under air-dried conditions.

The mineral composition of the MF1 marl from H1 Well is similar to that of D1 Well, with a notable reduction in clay mineral content (Fig 5). The calcite content is significantly higher, while quartz and dolomite contents are lower. Clay mineral content is comparatively reduced, with talc, kaolinite, chlorite, and illite/smectite present in lower amounts, and no sepiolite detected. The calcite content ranges from 41.2% to 97.4% (average 83.0%), quartz content ranges from 0.3% to 19.1% (average 7.2%), dolomite content ranges from 0% to 10.6% (average 3.4%), and clay content ranges from 0% to 22.9% (average 3.8%). The main clay minerals in H1 Well are talc, with a maximum content of 0% to 9.5% (average 2.3%), kaolinite with 0% to 0.9% (average 0.2%), chlorite with 0% to 0.5% (average 0.1%), and the illite/smectite mixed with 0% to 21.5% (average 3.6%).

### Trace and rare earth element composition

#### Composition characteristics of trace elements.

The trace element composition in the marl samples is generally characterized by enrichment in active elements such as Sr and Ba, moderate levels of U, Zr, and Rb, while the abundance of other trace elements is low, with most not exceeding 1 µg/g. Trace elements, including Rb, Sr, Ba, Zr, U, and others, were detected in carbonate rock core samples (Table 2). The trace element composition in the two wells shows significant variations. The D1 Well shows enrichment in Sr (average 1621 µg/g), Ba (average 39.81 µg/g), U (average 7.27 µg/g), Zr (average 6.33 µg/g), Rb (average 4.15 µg/g), Nb (average 1.60 µg/g), Th (average 1.38 µg/g), and Hf (average 0.19 µg/g). In contrast, H1 Well samples significantly show lower contents of Rb (average 1.76 µg/g), Sr (average 1031 µg/g), Ba (average 17.58 µg/g), Nb (average 0.62 µg/g), Zr (average 2.61 µg/g), Hf (average 0.08 µg/g), U (average 5.07 µg/g), Th (average 0.28 µg/g), and other trace elements than those of D1 Well.

**Table 2:** Trace element content and their ratios of MF1 strata in the D1 and H1 wells.

Sample	Rb	Sr	Ba	Nb	Zr	Hf	U	Th	Sr/Ba	Th/U
**D1**	1.05	1380	40.5	0.29	1.62	0.06	4.11	0.15	34.07	0.04
**D2**	3.52	4590	53.2	0.76	6.06	0.18	10.60	0.44	86.28	0.04
**D3**	2.30	3240	15.8	0.38	2.74	0.08	12.80	0.21	205.06	0.02
**D4**	1.09	2580	43.7	0.14	0.58	0.03	4.29	0.38	59.04	0.09
**D5**	2.93	876	30.2	1.75	6.76	0.19	3.58	0.53	29.01	0.15
**D6**	2.15	868	11.8	0.96	4.22	0.13	3.23	0.26	73.56	0.08
**D7**	4.54	733	51.9	1.08	5.28	0.19	5.04	4.51	14.12	0.89
**D8**	3.37	608	19.9	0.98	5.29	0.16	4.16	1.46	30.55	0.35
**D9**	17.40	449	79.5	9.43	29.20	0.82	21.10	2.91	5.65	0.14
**D10**	3.13	882	51.6	0.2	1.54	0.06	3.79	2.59	17.09	0.68
**H1**	0.90	1139	15.0	0.62	2.62	0.07	4.35	0.20	75.93	0.05
**H2**	0.95	1160	11.3	0.62	2.18	0.06	6.23	0.16	102.65	0.03
**H3**	0.96	934	12.9	0.49	1.73	0.05	7.46	0.15	72.40	0.02
**H4**	5.08	1562	42.5	1.37	5.23	0.15	5.07	0.70	36.75	0.14
**H5**	0.59	884	20.6	0.21	1.58	0.05	6.01	0.13	42.91	0.02
**H6**	2.05	606	23.1	0.43	2.35	0.08	1.27	0.35	26.23	0.28

Units: μg/g (micrograms per gram) for all elements.

#### Composition characteristics of rare earth elements.

The distribution patterns of rare earth elements (REE) in the MF1 carbonate rocks were standardized to the Post-Archaean Australian Shales (PAAS) to account for element parity effects and highlight differentiation [30]. The total rare earth element content (∑REE) refers to the sum of 14 rare earth elements including the light rare earth elements (LREE: La-Eu) and heavy rare earth elements (HREE: Gd ~ Lu). In H1 Well, the ∑REE values range from 4.050 µg/g to 10.715 µg/g (Table 3), with an average of 5.778 µg/g. The total REE content (∑REE) in D1 Well samples is 4.212 µg/g to 76.172 µg/g (average 31.270 µg/g), higher than in that in H1 Well. In general, the rare earth element composition of carbonate rocks from two wells is similar to the pattern of marine limestone [31–33], showing relative loss of light rare earth elements, and significant high Y/Ho ratio. The Ce anomalies in two wells are mostly negative, with δCe values ranging from 0.36 to 1.03, averaging 0.86.

**Table 3:** Rare earth element content in the MF1 marl of D1 and H1 wells.

Sample	La	Ce	Pr	Nd	Sm	Eu	Gd	Tb	Dy	Y	Ho	Er	Tm	Yb	Lu	∑REE	Y/Ho	δCe	δEu	(La/Yb)_N_	(La/Sm)_N_	(Gd/Yb)_N_
**D1**	0.648	1.325	0.149	0.532	0.087	0.046	0.095	0.014	0.081	1.079	0.018	0.062	0.010	0.058	0.008	4.212	59.94	0.98	2.36	0.82	1.08	0.99
**D2**	1.171	2.599	0.290	1.101	0.181	0.050	0.170	0.023	0.121	1.019	0.026	0.087	0.014	0.092	0.014	6.958	39.19	1.03	1.34	0.94	0.94	1.12
**D3**	0.740	1.501	0.170	0.651	0.120	0.029	0.110	0.017	0.112	1.090	0.022	0.066	0.014	0.077	0.010	4.729	49.55	0.98	1.19	0.71	0.90	0.86
**D4**	2.289	4.331	0.520	1.902	0.431	0.131	0.461	0.082	0.490	2.942	0.099	0.299	0.052	0.360	0.054	14.443	29.72	0.92	1.37	0.47	0.77	0.77
**D5**	1.321	2.989	0.351	1.210	0.199	0.034	0.201	0.025	0.119	0.901	0.029	0.079	0.014	0.085	0.014	7.571	31.07	1.01	0.80	1.15	0.96	1.43
**D6**	0.841	1.601	0.202	0.740	0.140	0.041	0.130	0.024	0.151	1.150	0.033	0.130	0.026	0.224	0.046	5.479	34.85	0.90	1.43	0.28	0.87	0.35
**D7**	4.802	9.090	1.150	4.091	1.091	0.171	1.910	0.751	7.489	72.801	1.830	6.181	6.181	8.892	1.510	127.94	39.78	0.89	0.52	0.04	0.64	0.13
**D8**	2.201	4.799	0.838	3.242	1.261	0.086	1.101	0.302	2.121	15.902	0.411	1.360	1.360	2.402	0.381	37.767	38.69	0.79	0.34	0.07	0.25	0.28
**D9**	2.690	5.790	0.850	3.229	0.961	0.086	0.781	0.171	1.181	8.221	0.250	0.792	0.792	1.413	0.231	27.438	32.88	0.87	0.47	0.14	0.41	0.33
**D10**	6.469	6.421	2.509	9.761	3.419	0.181	2.621	0.670	4.480	29.602	0.810	2.503	2.503	3.641	0.582	76.172	36.55	0.36	0.28	0.13	0.27	0.44
**H1**	0.883	1.352	0.170	0.631	0.122	0.034	0.114	0.020	0.105	1.047	0.025	0.072	0.012	0.074	0.011	4.672	41.88	0.80	1.36	0.88	1.05	0.93
**H2**	0.978	1.440	0.172	0.622	0.111	0.033	0.098	0.017	0.099	1.112	0.023	0.071	0.012	0.070	0.011	4.869	48.35	0.80	1.49	1.03	1.28	0.85
**H3**	0.854	1.376	0.171	0.628	0.115	0.029	0.085	0.013	0.070	0.583	0.016	0.043	0.008	0.051	0.008	4.050	36.44	0.83	1.38	1.24	1.08	1.01
**H4**	1.993	3.353	0.419	1.586	0.304	0.051	0.254	0.044	0.238	2.040	0.053	0.154	0.027	0.170	0.029	10.715	38.49	0.85	0.86	0.87	0.95	0.90
**H5**	0.803	1.287	0.155	0.589	0.120	0.031	0.114	0.019	0.106	1.048	0.023	0.070	0.012	0.070	0.012	4.459	45.57	0.84	1.25	0.85	0.97	0.99
**H6**	1.079	1.964	0.241	0.908	0.168	0.025	0.136	0.023	0.122	1.020	0.027	0.079	0.014	0.082	0.015	5.903	37.78	0.89	0.78	0.97	0.93	1.00

Units: μg/g (micrograms per gram) for all elements; δCe = 2Ce_N_/ (La_N_ + Pr_N_); δEu = 2Eu_N_/ (Sm_N_ + Gd_N_).

La and Yb serve as index elements for LREE and HREE, respectively, and their ratio (La/Yb)_N_ reflects the differentiation degree between light and heavy rare earth elements [34]. The (La/Yb)_N_ ratio in D1 Well samples ranges from 0.04 to 1.15 (avg. 0.47), suggesting strong LREE fractionation with HREE-enriched signatures, as evidenced by a pronounced left-leaning REE pattern. In contrast, the (La/Yb)_N_ ratio in H1 Well samples ranges from 0.85 to 1.24 (avg. 0.97), indicating moderate LREE enrichment characterized by a gentle left-inclined distribution trend. Additionally, the (La/Sm)_N_ ratio in D1 Well samples ranges from 0.25 to 1.08, averaging 0.71, indicating low differentiation of light rare earth elements with some loss of LREE. The (Gd/Yb)_N_ ratio ranges from 0.13 to 1.43, averaging 0.67, showing strong enrichment in heavy rare earth elements. In H1 Well, the (La/Sm)_N_ ratio ranges from 0.93 to 1.28, averaging 1.04, reflecting low fractionation and only slight loss of LREE. The (Gd/Yb)_N_ ratio ranges from 0.85 to 1.01, averaging 0.95, indicating low fractionation and slight enrichment of HREE.

The δEu values in D1 Well carbonate samples show a wide range from 0.28 to 2.36 (average 1.01), half of which exhibit negative Eu anomalies with the other half positive Eu anomalies. In H1 Well, two samples show negative Eu anomalies, while four samples show positive anomalies, with δEu values ranging from 0.78 to 1.49, averaging 1.19.

#### In-situ LA-ICP-MS result.

In-situ LA-ICP-MS analyses from six targeted locations (encompassing major oxides, trace elements, and rare earth elements) are compiled in Table 4, with their corresponding spatial positions delineated in Figs 2 and 3. The major and trace elements such as Al (0.48–3340.72 μg/g), Mg (2797.22–25389.51 μg/g), Si (71.86–32283.37 μg/g), S (328.67–1696.84 μg/g), Fe (0.01–2066.38 μg/g), and Sr (69.03–5310.13 μg/g) are relatively enriched, but their concentrations vary significantly among the samples. Based on the concentration levels of elements (S, Ti, Fe, Ba, Mo, Se, U, and Th) measured at six sampling spots, the samples can be clustered into two distinct groups: one exhibiting low elemental concentrations (D1-2, D2-1, and D9-1) and the other displaying significantly elevated concentrations (D1-1, D3-1, and D9-2). Similarly, the rare earth element (REE) characteristics also indicate two categories. The first group, characterized by positive Eu anomalies, includes D1-1 (δEu = 1.12), D3-1 (δEu = 1.12), and D9-2 (δEu = 1.10). The remaining three samples exhibit negative Eu anomalies, with values ranging from 0.72 to 0.85. Overall, the total rare earth content of the six samples is not high, ranging from 4.748 to 24.193 μg/g. The REE distribution patterns exhibit flat profiles, while samples D3-1 and D2-1 demonstrate a slight left-inclined pattern indicating light rare earth element (LREE) depletion. Overall, the six samples exhibit negative δCe anomalies (0.98–1.09).

**Table 4:** Element characteristics of LA-ICP-MS analysis result of MF1 marls.

Sample	Al	Mg	Si	S	Ti	Mn	Fe	Sr	Ba	Mo	Se	U	Th	Al/(Al + Fe + Mn)	Th/U	Sr/Ba						
**D1-1**	8.92	4401.71	10695.01	571.39	12.51	8.5	245.8	5310.13	0.75	3.21	87	7.29	0.08	0.03	0.01	7080.17						
**D1-2**	124.29	2797.22	2695.44	432.71	0.62	9.1	53.73	2599.48	14.39	0.2	0	0.8	0.75	0.66	0.94	180.64						
**D2-1**	0.48	11375.27	71.86	328.67	0.01	0	0.01	69.03	15.17	0.07	0	0.46	0.48	0.98	1.04	4.55						
**D3-1**	887.28	18999.15	32283.37	524.23	55.3	6.89	1233.47	3373.68	7.55	2.18	56.31	13.97	0.18	0.42	0.01	446.85						
**D9-1**	3340.72	25389.51	2454.08	701.45	0.01	8.29	56.38	1832.04	33.19	0.87	0	1.74	0.85	0.98	0.49	55.20						
**D9-2**	411.29	8798.06	6482.12	1696.84	50.09	47.1	2066.38	2604.87	7.5	43.75	65.72	4.69	0.56	0.16	0.12	347.32						
**Sample**	**La**	**Ce**	**Pr**	**Nd**	**Sm**	**Eu**	**Gd**	**Tb**	**Dy**	**Y**	**Ho**	**Er**	**Tm**	**Yb**	**Lu**	**∑REE**	**Y/Ho**	**δCe**	**δEu**	**(La/Yb)**_**N**_	**(La/Sm)**_**N**_	**(Gd/Yb)**_**N**_
**D1-1**	3.283	6.148	0.592	2.245	0.677	0.175	0.783	0.125	0.726	4.761	0.150	0.312	0.030	0.215	0.024	20.246	31.74	1.01	1.12	1.13	0.70	2.20
**D1-2**	3.043	5.234	0.481	1.705	0.489	0.095	0.555	0.066	0.492	2.929	0.084	0.242	0.029	0.169	0.019	15.632	34.87	0.98	0.85	1.33	0.90	1.99
**D2-1**	1.149	2.316	0.224	0.920	0.169	0.026	0.168	0.033	0.192	1.962	0.056	0.177	0.017	0.199	0.017	7.625	35.04	1.05	0.72	0.43	0.99	0.51
**D3-1**	0.689	1.537	0.155	0.511	0.102	0.024	0.099	0.013	0.113	1.273	0.030	0.078	0.016	0.084	0.024	4.748	42.43	1.09	1.12	0.61	0.98	0.71
**D9-1**	4.211	8.541	0.902	3.355	0.660	0.098	0.469	0.058	0.443	4.829	0.078	0.268	0.033	0.210	0.038	24.193	61.91	1.01	0.83	1.48	0.93	1.35
**D9-2**	2.562	4.817	0.452	1.579	0.357	0.081	0.335	0.079	0.355	3.907	0.079	0.209	0.036	0.198	0.042	15.088	49.46	1.02	1.10	0.96	1.04	1.02

Units: μg/g (micrograms per gram) for all elements.

## Discussion

### Genesis of MCMs in MF1 strata

**Fig 6:**
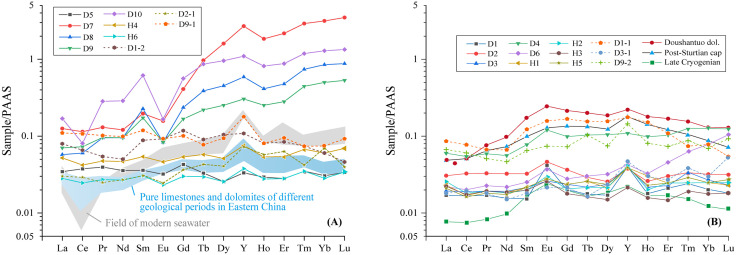
PAAS-normalized rare earth element (REE) distribution patterns of two types of MF1 MCMs. The sixteen REE curves were obtained from ten samples (D1 to D10) analyzed by whole-rock ICP-MS, while six points (D1-1, D1-2, D2-1, D3-1, D9-1 and D9-2) were analyed in situ by LA-ICP-MS. REE-Y data of modern seawater (Bolhar et al., 2004), Pure limestones and dolomites of different geological periods in Eastern China [43], Doushantuo dolomite [33], Post-Sturtian cap carbonates [42], and Late Cryogenianmarine carbonates [41] are shown for comparison.

Different depositional or diagenetic fluids have distinct REE distribution patterns and characteristics. Fluids under different oxidation and reduction conditions exhibit varying degrees of Ce and Eu anomalies [35–37]. After PAAS standardization, the REE content of 16 MF1 samples from two wells shows relatively consistent distribution patterns, which align with the characteristics of marine carbonate REEs, such as light REE depletion, positive Y anomaly, and negative Ce anomaly. The total REE content in both wells is extremely low, with ∑REE ranging from 4.212 to 76.172 μg/g, significantly lower than the ∑REE value of 184.8 μg/g for Post Archean Australian Shale (PAAS), indicating that the MF1 marl experienced different degrees of contamination from terrigenous clastic material during deposition [21]. Overall, the total REE content in D1 well is higher than that in H1 well, with clay minerals such as sepiolite and talc also more abundant in D1 well. The Y/Ho ratio of terrigenous sediments ranges from 26 to 28, the modern seawater is 44, and for marine sediments, it ranges from 44 to 72 [36]. The Y/Ho ratio in D1 well ranges from 29.72 to 59.94 (average 39.22), and in well H1, it ranges from 36.44 to 48.35 (average 41.42), indicating that the MF1 marl were influenced by terrigenous clastic contamination. Based on δEu anomalies [38], the MCMs can be categorized into two types: one type shows a negative δEu anomaly, higher total REE content, and an enrichment of heavy REEs, with the REE distribution curve showing a leftward or flat pattern. This type includes samples D5, D7, D8, D9, and D10 from D1 well, and H4 and H6 from H1 well (Fig 6A). These samples are characterized by low Sr and U contents, low Sr/Ba ratios (5.65–36.75), and high Th/U ratios (0.14–0.89), significantly influenced by terrigenous input, with more clay minerals in the MF1 marl, and classified as sedimentary-type MCMs [39,40]. The other type, represented by nine samples (e.g., D1 well: D1, D2, D3, D4, D6; H1 well: H1, H2, H3, H5), shows lower total rare earth element (REE) contents. The REE depletion and positive Y anomalies confirm that the marl formed in a marine environment [31], while variations in total REE content reflect changes in sedimentary water depth [32]. The in-situ REE characteristics of sepiolite align with those of the marlstone, exhibiting typical positive Eu anomalies and LREE depletion, indicating a mixed seawater-hydrothermal origin for REE distribution. As shown in Fig 6, Late Cryogenian marine carbonates [41], Doushantuo dolomite [33], and Post-Sturtian cap carbonates [42] demonstrate significant hydrothermal influence during deposition. The other type of MCMs in this study displays relatively flat REE distribution patterns with LREE depletion and positive Eu anomalies, enriched in Sr and U, high Sr/Ba ratios (34.07–205.06), and low Th/U ratios (0.02–0.09), suggesting a hydrothermal origin for the sepiolite (Fig 6B).

### Sedimentary MCMs

The mid-Permian period is a peak in biogenic siliceous deposition, coinciding with the Permian Siliceous Sedimentary Event [44–47]. During this period, volcanic activities were frequent, and volcanic tuffaceous materials entered the ocean [48,49]. The MCMs can only precipitate in seawater environments rich in Mg, Si, Al with high pH, and low temperature [21,50,51]. Layered, striped, and massive sepiolite demonstrated a sedimentary origin, and formed either through direct chemical precipitation from seawater as the concentrations of dissolved Si and Mg increase or by syn-sedimentary diagenetic replacement of bioclasts. Sepiolite and talc typically form in specific sedimentary environments. Sepiolite commonly precipitates in shallow, evaporitic settings characterized by low-energy conditions and abundant magnesium and silica sources. Talc formation occurs under conditions with an excess of magnesium-rich fluids and silica, typically within sedimentary basins featuring particular hydrogeological conditions, such as seawater terraces and lagoons. In contrast, calcite and dolomite are generally associated with carbonate-rich sedimentary environments, such as shallow marine platforms. The low content of these carbonate minerals in sample D9 indicates that the sedimentation environment had limited carbonate input, potentially due to its distance from carbonate-producing organisms or an unfavorable chemical milieu for carbonate precipitation.

**Fig 7:**
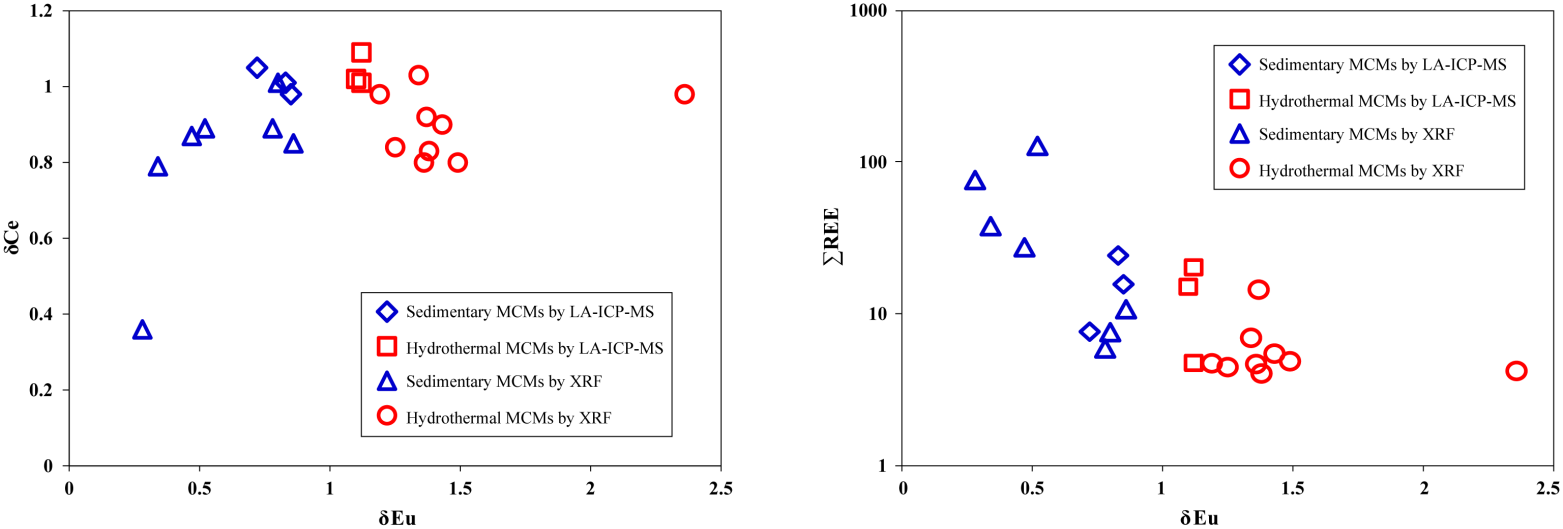
Cross-plots of Eu anomalies vs. Ce anomalies (Left) and Eu anomalies vs. **∑REE (Right)**.

Apart from the seven previously mentioned samples, three in-situ point samples (D1-2, D2-1, D9-1) also exhibit characteristics of sedimentary MCMs based on their in-situ element profiles (Table 4), showing low levels of hydrothermal-related elements such as low Ti, S, relatively low Si, Sr, Mo, and Se, with low Sr/Ba ratios (4.55–180.64) and high Th/U ratios (0.49–1.04). The D2-1 sample is a massive sepiolite block (Fig 2B). The D9-1 sample shows sepiolite replacing coralline algae (Fig 2C), and D1-2 sample represents MCMs within bioclast cavities (Fig 2F). The Y/Ho ratio is less than 44, indicating the presence of terrigenous material admixture [52]. The (La/Yb)_N_ value is 0.43, reflecting heavy REE enrichment, suggesting influence from marine sedimentation processes [39,53,54]. The three MCMs samples exhibit distinct negative δEu anomalies, and these distribution curves align closely with the overall REE patterns observed in the seven whole-rock samples (Figs 6A and 7). This consistency suggests that the geochemical signatures of MCMs formation likely inherited features from the whole-rock system, where the negative Eu anomalies in carbonate rocks may be influenced by recycled crustal materials, such as clay-rich sediments [55]. The REE distribution patterns of marl and MCMs likely inherit crust-derived negative Eu-anomalies, reflecting terrigenous clastic inputs that modified REE signatures in sedimentary carbonates [32].

#### Hydrothermal MCMs.

Hydrothermal fluids generally exhibit significantly higher ∑REE values than seawater, characterized by light REE enrichment and a pronounced positive Eu anomaly in their distribution patterns [56]. The nine samples of D1, D2, D3, D4, D6, H1, H2, H3, and H5 show positive Eu anomaly from 1.19 to 2.36. Through microscopy and in-situ ICP-MS analysis, the sepiolite replacing bioclasts, as well as massive MCMs, shows evidence of hydrothermal influence. In-situ point samples D1-1, D3-1, and D9-2 exhibit hydrothermal-related elemental characteristics such as high Ti, S, relatively high Si, Sr, Mo, and Se, high Sr/Ba ratios (347.32–7080.17), and low Th/U ratios (0.01–0.12). In marine sediments, the Al/(Al + Fe + Mn) ratio is also used to measure the contribution of hydrothermal activity to sediment deposition [57]. For instance, the brown-yellow to brown-black MCMs in sample D9-2 (Fig 2E) shows a distinct positive Eu anomaly (Fig 7) and an Al/(Al + Fe + Mn) ratio of 0.16. The MCMs of sample D1-1 exhibits an Al/(Al + Fe + Mn) ratio of 0.03, also indicative of typical hydrothermal, suggesting that even MCMs within bioclast cavities has been affected by hydrothermal activity (Fig 2F). The Al/(Al + Fe + Mn) value less than 0.35 is a typical characteristic of hydrothermal deposition, with lower values indicating stronger hydrothermal influence [58].

**Fig 8:**
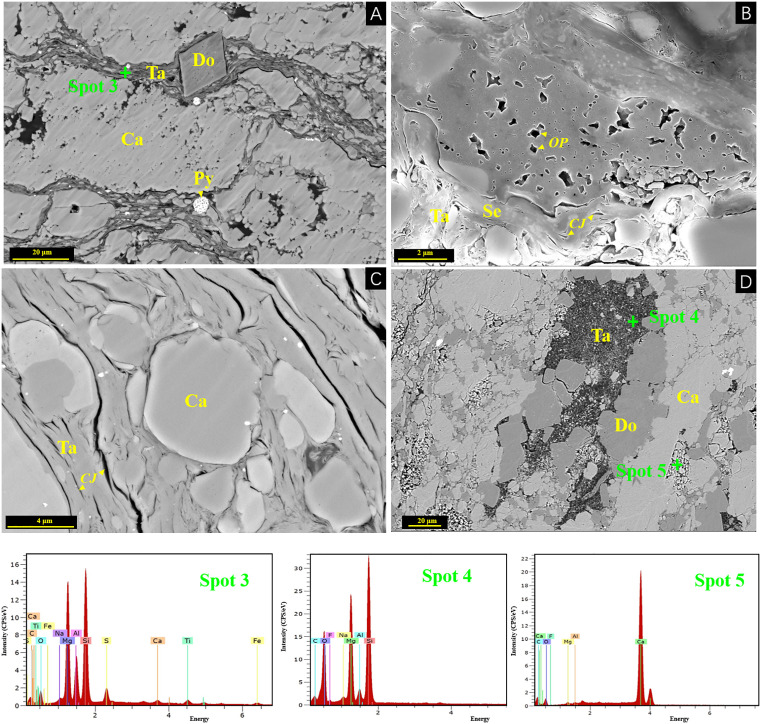
Reservoir construction of MF1 MCMs in the H1 well. **A.** Organic pores (OP), 4251.54m; **B.** Talc contraction joint (CJ), 4251.61m; **C.** Sepiolite transforms into talc (Ta), and dolomite (Do), 4253.77m; **D.** Dolomite crystals and strawberry pyrite in the dissolution soluble, 4240.26m. Elemental characteristics of Spot 3, Spot 4, and Spot 5 are detailed in S1 Table.

Frequent volcanic activity during the Permian provides a geological background for regional hydrothermal processes [49]. The Maokou sedimentation coincided with the Emeishan basalt eruptions [26], with well-developed syn-sedimentary faults along both the interior and margins of Sichuan Basin [59,60]. Hydrothermal fluids ascended along these faults and fractures, influencing the mineral and elemental composition of the MF1 reservoir. These fluids then interacted with biogenic high-magnesium sedimentary components, such as spots D2-1 and D9-1, extracting the precursor materials required for hydrothermal MCMs (Table 4). Interlayer fractures in the MF1 marl and intercrystalline pores served as hydrothermal conduits. Microscopic observations provide robust support for this mechanism. The presence of dissolution rims around bioclasts indicates interactions and dissolution processes induced by hydrothermal fluids. The replacement process was further evidenced by the preservation of original bioclast shapes within MCMs, suggesting a direct substitution. Additionally, the occurrence of fine-grained MCMs within intercrystalline pores implies the transport and deposition of elements via these pathways. Notably, some large sepiolite and quartz grains are preserved within the intercrystalline dissolution pores of calcite surrounding bioclasts (Figs 3D and 3E), which contradicts a purely sedimentary origin, as such an origin would typically produce a more uniform distribution of these minerals. Furthermore, fluid flow features were observed, where MCMs are seen filling between calcite grains (Fig 8A), accompanied by spectral data indicating elevated levels of hydrothermal elements such as sulfur and titanium (Spot 3). Thus, the magnesium-rich MF1 marl were in contact with silica-rich hydrothermal fluids, resulting in the formation of hydrothermal-type MCMs through the replacement of bioclasts or other clay minerals under hydrothermal activities.

### Reservoir implications

Sedimentary and hydrothermal MCMs show distinct textural characteristics in their occurrences. For sedimentary MCMs, they generally occur in two forms. One is the replacement of bioclasts (Figs 2B and 2C), and currently, talc is the dominant mineral in this form. This indicates a sedimentary-related diagenetic process where the original bioclasts are gradually replaced by talc under sedimentary conditions. The other form is that they are distributed in a granular and star-dotted pattern (Fig 3H) within calcite pores. This distribution is related to the sedimentary environment where MCMs are deposited and fill the pores of calcite during the sedimentation and diagenesis processes. Hydrothermal MCMs also occur in two forms. One is also the replacement of bioclasts (Figs 3D and 3E), but sepiolite is the main mineral. This implies that the diagenetic fluids likely existed in a relatively closed system, constraining clay mineral transformation. It is also possible that this is related to the stable crystal structure of hydrothermal sepiolite and its relatively high mineral transformation temperature [61]. The other form is that they are distributed in stylolites and dissolution fractures and show a flow structure (Fig 8A). The uneven distribution and the presence of a flow structure are typical features of hydrothermal fluids. The hydrothermal fluids carrying MCMs flow through these fractures and stylolites, and the minerals are precipitated during the cooling and chemical reaction processes. These differences in occurrence forms and distribution patterns clearly distinguish sedimentary and hydrothermal MCMs.

The MCMs play a significant positive role in the formation of the MF1 carbonate reservoir. Due to the strong adsorption of sepiolite [21], a large amount of organic matter was adsorbed during the deposition of sepiolite-bearing marl, and a large number of organic matter pores and clay micropores were formed during the maturation and evolution of organic matter in the later diagenetic process (Fig 8B). During the diagenesis process, sepiolite gradually transforms into stevensite and talc [12,62]. Although the critical temperature for the mineral transformation of sepiolite is relatively low, for example, the critical temperature for the transformation of sepiolite to stevensite is 120–130°C [62], the MF1 paleotemperatures historically experienced in the central and southern Sichuan Basin were all much higher than 120°C [10]. Consequently, the present-day proportion of sepiolite within the MF1 strata should be low or negligible (Table 1). However, analyses of Sample D9 and previous data [12] indicate that a substantial proportion of sepiolite is still retained in the MCMs of the MF1. This suggests that, in addition to being affected by temperature and burial time [63], it is also controlled by complex diagenetic conditions and processes such as formation pressure and pore-fluid connectivity. Sepiolite is mostly fibrous aggregates, which are feathery after aggregation, while talc is mostly flaky and has rich contraction joints (Fig 8C). These diagenetic contraction joints can be used as effective reservoir space for the dense MF1 strata.

The crystal of talc is smaller than that of the original sepiolite crystal [12], and intergranular pores are more developed as the burial depth increases and the transformation degree rises (Fig 8D). Meanwhile, the conversion process of sepiolite to talc will release a large amount of magnesium and silicon ions, and surrounding calcite can be dolomitized (Fig 8D) with intercrystalline pores (Fig 8A), which helps to increase the reservoir porosity [10]. The formed granular quartz, likely resulting from hydrothermal mineralization, exhibits high compressive resistance (Fig 8D) and can further enhance the reservoir’s physical properties as a brittle mineral. The sepiolite is mostly associated with talc and quartz (Fig 4G). The MF1 marl exhibits relatively low porosity, typically below 4%, reflecting its dense lithology [9]. However, the presence of sepiolite within this formation significantly enhances reservoir quality by creating secondary porosity through mineralogical transformations. For instance, diagenetic processes such as the conversion of sepiolite to talc generate shrinkage joints and micro-fractures, which serve as effective storage spaces for hydrocarbons. Notably, in the eastern Sichuan Basin, commercial oil and gas production from MF1 well sections are closely associated with sepiolite-rich intervals, highlighting its critical role in improving reservoir permeability and connectivity [21].

## Conclusions

The MF1 strata in the Sichuan Basin contain MCMs in three primary forms: replaced biological shell or cavity, agglomerate or lenticular, and bedding distribution. These MCMs varieties can be classified into sedimentary and hydrothermal types. Sedimentary MCMs from terrigenous clastic input likely formed through chemical precipitation from seawater rich in Si and Mg or by syn-sedimentary diagenetic replacement of bioclasts. In contrast, hydrothermal MCMs likely formed via metasomatism driven by siliceous hydrothermal fluids interacting with magnesium-rich carbonate rocks. The development of organic matter pores, talc contraction pores, and increased dolomitization and quartz formation due to sepiolite diagenesis significantly enhances the physical properties of the MF1 marl reservoir. Therefore, the MF1 strata with well-developed MCMs are considered the sweet spot for oil and gas exploration in the Sichuan Basin.

## Supporting information

S1 Table EDS quantitative chemical data for all analyzed spots. (DOCX)

## References

[1] Jarvie DM, Hill RJ, Ruble TE, Pollastro RM. Unconventional shale-gas systems: The Mississippian Barnett Shale of north-central Texas as one model for thermogenic shale-gas assessment. Bulletin. 2007;91(4):475–99. doi: 10.1306/12190606068

[2] Slatt RM, O’Brien NR. Pore types in the Barnett and Woodford gas shales: Contribution to understanding gas storage and migration pathways in fine-grained rocks. Bulletin. 2011;95(12):2017–30. doi: 10.1306/03301110145

[3] Zou CN, Yang Z, Tao SZ, Yuan XJ, Zhu RK, Hou LH, et al. Continuous hydrocarbon accumulation over a large area as a distinguishing characteristic of unconventional petroleum: The Ordos Basin, North-Central China. Earth-Science Reviews. 2013;126:358–69. doi: 10.1016/j.earscirev.2013.08.006

[4] Hollis C, Vahrenkamp V, Tull S, Mookerjee A, Taberner C, Huang Y. Pore system characterisation in heterogeneous carbonates: An alternative approach to widely-used rock-typing methodologies. Mar Pet Geol. 2010;27(4):772–93.

[5] Hammes U, Krause M, Mutti M. Unconventional reservoir potential of the upper Permian Zechstein Group: a slope to basin sequence stratigraphic and sedimentological evaluation of carbonates and organic-rich mudrocks, Northern Germany. Environ Earth Sci. 2013;70(8):3797–816. doi: 10.1007/s12665-013-2724-1

[6] Radwan AE, Trippetta F, Kassem AA, Kania M. Multi-scale characterization of unconventional tight carbonate reservoir: Insights from October oil filed, Gulf of Suez rift basin, Egypt. Journal of Petroleum Science and Engineering. 2021;197:107968. doi: 10.1016/j.petrol.2020.107968

[7] Yao W, Xu J, Xia WQ, Wang Q, Rao D, Chen QL. A characteristic analysis between acidolysis gas and absorbed gas and its application to gas-source correlation in Mao 1 Member, Fuling area, Sichuan Basin. Nat Gas Ind. 2019;39(06):45–50.

[8] Zhang PX, He XP, Gao QF, Gao YQ, Sun B, Cai X. Geological characteristics and enrichment pattern of Permian Mao 1 Member shale gas reservoirs at the southeastern margin of Sichuan Basin. Oil Gas Geol. 2021;42(1):146–57.

[9] Jiang QC, Wang ZC, Su W, Huang SP, Zeng FY, Feng Z. Accumulation conditions and favorable exploration orientation of unconventional natural gas in the marl source rock of the first member of the Middle Permian Maokou Formation, Sichuan Basin. China Pet Explor. 2021;26(06):82–97.

[10] Jiang Q, Wang W, Lyu Q. Characteristics and Controlling Factors of Tight Marl Reservoirs with an Eyelid-Shaped Structure of the First Member of the Deep Maokou Formation in Eastern Sichuan. Energies. 2023;16(5):2353. doi: 10.3390/en16052353

[11] Su CP, Li F, Tan XC, Gong QL, Zeng K, Tang H. Recognition of diagenetic contribution to the formation of limestone-marl alternations: A case study from Permian of South China. Mar Pet Geol. 2020;111:765–85.

[12] Song JM, Wang JR, Liu SG, Li ZW, Luo P, Jiang QC. Types, composition and diagenetic evolution of authigenic clay minerals in argillaceous limestone of sepiolite-bearing strata: A case study of Mao-1 Member of Middle Permian Maokou Formation, eastern Sichuan Basin, SW China. Petroleum Exploration and Development. 2024;51(2):351–63.

[13] Liu XT, Yan JX, Ma ZX, Ma ZX, Xue WQ. Origination of Limestone-Marl Alternations from Qixia Formation of South China. Earth Sci. 2014;39(2):155–64.

[14] Birsoy R. Formation of Sepiolite-Palygorskite and Related Minerals from Solution. Clays and clay miner. 2002;50(6):736–45. doi: 10.1346/000986002762090263

[15] Westphal H, Head MJ, Munnecke A. Differential diagenesis of rhythmic limestone alternations supported by palynological evidence. J Sediment Res. 2000;70(3):715–25.

[16] Ricken W. Epicontinental marl-limestone alternations: event deposition and diagenetic bedding (upper Jurassic, southwest Germany). Berlin: Springer Verlag. 1985.

[17] Tateo F, Sabbadini R, Morandi N. Palygorskite and sepiolite occurrence in Pliocene lake deposits along the River Nile: evidence of an arid climate. Journal of African Earth Sciences. 2000;31(3–4):633–45. doi: 10.1016/s0899-5362(00)80011-1

[18] Munnecke A, Westphal H. Shallow-water aragonite recorded in bundles of limestone–marl alternations—the Upper Jurassic of SW Germany. Sedimentary Geology. 2004;164(3–4):191–202. doi: 10.1016/j.sedgeo.2003.10.002

[19] Galán E, Singer A. Developments in palygorskite-sepiolite research: A new outlook on these nanomaterials. Amsterdam: Elsevier. 2011.

[20] Pozo M, Calvo JP, Pozo E, Moreno Á. Genetic constraints on crystallinity, thermal behaviour and surface area of sepiolite from the Cerro de los Batallones deposit (Madrid Basin, Spain). Applied Clay Science. 2014;91–92:30–45. doi: 10.1016/j.clay.2014.02.005

[21] Song JM, Jiang QC, Liu SG, Jin X, Fan JP, Li ZW. Paleoenvironment and sedimentary significances of sepiolite-containing succession in the first member of Middle Permian Maokou Formation, Sichuan Basin. Acta Pet Sin. 2024;45(6):914–31.

[22] Draidia S, El Ouahabi M, Daoudi L, Havenith H-B, Fagel N. Occurrences and genesis of palygorskite/sepiolite and associated minerals in the Barzaman formation, United Arab Emirates. Clay miner. 2016;51(5):763–79. doi: 10.1180/claymin.2016.051.5.06

[23] Liu S, Yang Y, Deng B, Zhong Y, Wen L, Sun W, et al. Tectonic evolution of the Sichuan Basin, Southwest China. Earth-Science Reviews. 2021;213:103470. doi: 10.1016/j.earscirev.2020.103470

[24] Hao Y, Yao QY, Tian H, Gu MF, She M, Wang Y. Sedimentary characteristics and reservoir-controlling factors of the Permian Maokou Formation in Sichuan Basin. Mar Origin Pet Geol. 2020;25(03):202–9.

[25] Xiao D, Tan XC, Xi AH, Liu H, Shan SJ, Xia JW. An inland facies-controlled eogenetic karst of the carbonate reservoir in the Middle Permian Maokou Formation, southern Sichuan Basin, SW China. Mar Pet Geol. 2016;72:218–33.

[26] Su W, Hu S, Jiang Q, Zhang J, Huang S, Jiang H, et al. Sedimentary responses to the Dongwu movement and the influence of the Emeishan mantle plume in Sichuan Basin, Southwest China: significance for petroleum geology. Carbonates Evaporites. 2020;35(4). doi: 10.1007/s13146-020-00638-w

[27] Kershaw S, Crasquin S, Li Y, Collin P-Y, Forel M-B, Mu X, et al. Microbialites and global environmental change across the Permian-Triassic boundary: a synthesis. Geobiology. 2012;10(1):25–47. doi: 10.1111/j.1472-4669.2011.00302.x 22077322

[28] Lehrmann DJ, Payne JL, Felix SV, Dillett PM, Wang H, Yu Y, et al. Permian-Triassic Boundary Sections from Shallow-Marine Carbonate Platforms of the Nanpanjiang Basin, South China: Implications for Oceanic Conditions Associated with the End-Permian Extinction and Its Aftermath. PALAIOS. 2003;18(2):138–52. doi: 10.1669/0883-1351(2003)18<138:pbsfsc>2.0.co;2

[29] Haq BU, Schutter SR. A chronology of Paleozoic sea-level changes. Science. 2008;322(5898):64–8. doi: 10.1126/science.1161648 18832639

[30] Mclennan SM. Rare earth elements in sedimentary rocks; influence of provenance and sedimentary processes. Rev Mineral Geochem. 1989;21(1):169–200.

[31] Bolhar R, Kamber BS, Moorbath S, Fedo CM, Whitehouse MJ. Characterisation of early Archaean chemical sediments by trace element signatures. Earth and Planetary Science Letters. 2004;222(1):43–60. doi: 10.1016/j.epsl.2004.02.016

[32] Frimmel HE. Trace element distribution in Neoproterozoic carbonates as palaeoenvironmental indicator. Chem Geol. 2009;258(3/4):338–53.

[33] Ren M, Li R. Rare earth element signatures of Doushantuo cap dolostones capture an increase in oxygen in the anoxic Ediacaran ocean. Sedimentary Geology. 2023;446:106343. doi: 10.1016/j.sedgeo.2023.106343

[34] Wiche O, Zertani V, Hentschel W, Achtziger R, Midula P. Germanium and rare earth elements in topsoil and soil-grown plants on different land use types in the mining area of Freiberg (Germany). J Geochem Explor. 2017;175:120–9.

[35] Zhang J, Nozaki Y. Rare earth elements and yttrium in seawater: ICP-MS determinations in the East Caroline, Coral Sea, and South Fiji basins of the western South Pacific Ocean. Geochimica et Cosmochimica Acta. 1996;60(23):4631–44. doi: 10.1016/s0016-7037(96)00276-1

[36] Webb GE, Kamber BS. Rare earth elements in Holocene reefal microbialites: a new shallow seawater proxy. Geochimica et Cosmochimica Acta. 2000;64(9):1557–65. doi: 10.1016/s0016-7037(99)00400-7

[37] De Carlo EH, Wen X-Y, Irving M. The Influence of Redox Reactions on the Uptake of Dissolved Ce by Suspended Fe and Mn Oxide Particles. Aquatic Geochemistry. 1997;3(4):357–89. doi: 10.1023/a:1009664626181

[38] Lawrence MG, Greig A, Collerson KD, Kamber BS. Rare earth element and yttrium variability in South East Queensland waterways. Aquat Geochem. 2006;12(1):39–72.

[39] Byrne RH, Kim K-H. Rare earth element scavenging in seawater. Geochimica et Cosmochimica Acta. 1990;54(10):2645–56. doi: 10.1016/0016-7037(90)90002-3

[40] Shields GA, Webb GE. Has the REE composition of seawater changed over geological time?. Chemical Geology. 2004;204(1–2):103–7. doi: 10.1016/j.chemgeo.2003.09.010

[41] Hood A van S, Wallace MW. Extreme ocean anoxia during the Late Cryogenian recorded in reefal carbonates of Southern Australia. Precambrian Research. 2015;261:96–111. doi: 10.1016/j.precamres.2015.02.008

[42] Vieira LC, Nédélec A, Fabre S, Trindade RIF, de Almeida RP. Aragonite crystal fans in Neoproterozoic cap carbonates: a case study from Brazil and implications for the post-snowball earth coastal environment. J Sediment Res. 2015;85:285–300.

[43] Liu QQ, Chi QH, Wang XQ, Zhou J, Liu HL, Liu DS. Distribution and influencing factors of rare earth elements in carbonate rocks along three continental scale transects in eastern China. Earth Sci Front. 2018;25(4):99–115.

[44] Lowenstein TK, Timofeeff MN, Brennan ST, Hardie LA, Demicco RV. Oscillations in Phanerozoic seawater chemistry: evidence from fluid inclusions. Science. 2001;294(5544):1086–8. doi: 10.1126/science.1064280 11691988

[45] Beauchamp B, Baud A. Growth and demise of Permian biogenic chert along northwest Pangea: evidence for end-Permian collapse of thermohaline circulation. Palaeogeography, Palaeoclimatology, Palaeoecology. 2002;184(1–2):37–63. doi: 10.1016/s0031-0182(02)00245-6

[46] Wang W, Garbelli C, Zhang F, Zheng Q, Zhang Y, Yuan D, et al. A high-resolution Middle to Late Permian paleotemperature curve reconstructed using oxygen isotopes of well-preserved brachiopod shells. Earth and Planetary Science Letters. 2020;540:116245. doi: 10.1016/j.epsl.2020.116245

[47] Gao X, Liu Q, Fang C, Guo Y. Origin and Formation Mechanism of the Late Permian Black Siliceous Rocks in the Lower Yangtze Region. ACS Omega. 2024;9(16):17848–59. doi: 10.1021/acsomega.3c08384 38680372 PMC11044223

[48] Macdonald FA, Swanson-Hysell NL, Park Y, Lisiecki L, Jagoutz O. Arc-continent collisions in the tropics set Earth’s climate state. Science. 2019;364(6436):181–4. doi: 10.1126/science.aav5300 30872536

[49] Huang H, Huyskens MH, Yin Q-Z, Cawood PA, Hou M, Yang J, et al. Eruptive tempo of Emeishan large igneous province, southwestern China and northern Vietnam: Relations to biotic crises and paleoclimate changes around the Guadalupian-Lopingian boundary. Geology. 2022;50(9):1083–7. doi: 10.1130/g50183.1

[50] Baldermann A, Mavromatis V, Frick PM, Dietzel M. Effect of aqueous Si/Mg ratio and pH on the nucleation and growth of sepiolite at 25 °C. Geochimica et Cosmochimica Acta. 2018;227:211–26. doi: 10.1016/j.gca.2018.02.027

[51] Tosca NJ, Masterson AL. Chemical controls on incipient Mg-silicate crystallization at 25°C: Implications for early and late diagenesis. Clay miner. 2014;49(2):165–94. doi: 10.1180/claymin.2014.049.2.03

[52] Bau M. Controls on the fractionation of isovalent trace elements in magmatic and aqueous systems: evidence from Y/Ho, Zr/Hf, and lanthanide tetrad effect. Contrib Mineral Petrol. 1996;123:323–33.

[53] Nothdurft LD, Webb GE, Kamber BS. Rare earth element geochemistry of Late Devonian reefal carbonates, Canning Basin, Western Australia: confirmation of a seawater REE proxy in ancient limestones. Geochimica et Cosmochimica Acta. 2004;68(2):263–83. doi: 10.1016/s0016-7037(03)00422-8

[54] Tostevin R, Shields GGA, Tarbuck GGM, He TC, Clarkson MO, Wood RA. Effective use of cerium anomalies as a redox proxy in carbonate-dominated marine settings. Chem Geol. 2016;438:146–62.

[55] Elderfield H, Upstill-Goddard R, Sholkovitz ER. The rare earth elements in rivers, estuaries, and coastal seas and their significance to the composition of ocean waters. Geochimica et Cosmochimica Acta. 1990;54(4):971–91. doi: 10.1016/0016-7037(90)90432-k

[56] Michard A, Albarède F. The REE content of some hydrothermal fluids. Chem Geol. 1986;55(1):51–60.

[57] Boström K, Peterson MNA. The origin of aluminum-poor ferromanganoan sediments in areas of high heat flow on the East Pacific Rise. Mar Geol. 1969;7(5):427–47.

[58] Adachi M, Yamamoto K, Sugisaki R. Hydrothermal chert and associated siliceous rocks from the northern Pacific their geological significance as indication of ocean ridge activity. Sediment Geol. 1986;47(1–2):125–48.

[59] Wang Z, Zhao W, Hu S, Xu A, Jiang Q, Jiang H, et al. Control of tectonic differentiation on the formation of large oil and gas fields in craton basins: A case study of Sinian–Triassic of the Sichuan Basin. Natural Gas Industry B. 2017;4(2):141–55. doi: 10.1016/j.ngib.2017.07.019

[60] Deng L, Yang J, Yan Q, Song B, Tang H, Xiang Z, et al. Rifting of the upper Yangtze carbonate platform: Insight from middle Permian carbonate olistostromes in the NE Sichuan basin, SW China. Journal of Asian Earth Sciences. 2024;260:105973. doi: 10.1016/j.jseaes.2023.105973

[61] Pozo M, Calvo J. An Overview of Authigenic Magnesian Clays. Minerals. 2018;8(11):520. doi: 10.3390/min8110520

[62] Cai Z, Li J, Chen H, Cong F, Wu N, Wang L, et al. Genesis of Mg-phyllosilicate occurrences in the Middle Permian marine successions of South China. Applied Clay Science. 2019;181:105242. doi: 10.1016/j.clay.2019.105242

[63] Otsuka R, Sakamoto T, Hara Y. Phase transformations of sepiolite under hydrothermal conditions. J Clay Sci Soc Japan. 1974;14(1):8–19.

