# Supplementary material for: Origin of Mg-rich clay minerals in the first member of Maokou Formation in the middle Permian in the Central and Southern Sichuan Basin (China) and their implications on supergene and hypogene fluid regimes
Source: PLoS One. 2025 Dec 5;20(12):e0338303. doi: 10.1371/journal.pone.0338303 (PMC12680178; doi:10.1371/journal.pone.0338303)
Supplement: S1 Table — (DOCX) [file pone.0338303.s001.docx]

| **S1 Table EDS quantitative chemical data for all analyzed spots** | | | | | | |
| --- | --- | --- | --- | --- | --- | --- |
| **Spot** | **Element** | **Atomic**  **Number** | **Unnormalized Concentration** | **Normalized Concentration** | **Atomic** | **Sigma** |
|  |  |  | wt.% | wt.% | at.% | wt.% |
| **Spot1** | Si | 14 | 17.83 | 39.53 | 33.17 | 0.77 |
|  | Mg | 12 | 11.99 | 26.58 | 25.77 | 0.66 |
|  | O | 8 | 9.22 | 20.44 | 30.11 | 1.16 |
|  | Al | 13 | 3.43 | 7.6 | 6.64 | 0.19 |
|  | S | 16 | 1.43 | 3.16 | 2.32 | 0.08 |
|  | Ca | 20 | 0.49 | 1.08 | 0.63 | 0.04 |
|  | Na | 11 | 0.41 | 0.91 | 0.94 | 0.05 |
|  | K | 19 | 0.32 | 0.7 | 0.42 | 0.04 |
|  | Total | | 45.11 | 100 | 100 |  |
| **Spot2** | Si | 14 | 23.71 | 40.12 | 31.43 | 1.02 |
|  | O | 8 | 20.01 | 33.86 | 46.56 | 2.68 |
|  | Mg | 12 | 10.90 | 18.44 | 16.69 | 0.61 |
|  | Ca | 20 | 3.03 | 5.13 | 2.82 | 0.14 |
|  | F | 9 | 0.86 | 1.45 | 1.68 | 0.25 |
|  | Al | 13 | 0.59 | 1.00 | 0.81 | 0.06 |
|  | Total | | 59.1 | 100 |  |  |
| **Spot3** | C | 6 | 17.32 | 25.58 | 41.12 | 3.41 |
|  | Si | 14 | 16.34 | 24.13 | 16.59 | 0.72 |
|  | Mg | 12 | 14.96 | 22.09 | 17.55 | 0.83 |
|  | O | 8 | 8.04 | 11.87 | 14.32 | 1.52 |
|  | Al | 13 | 6.28 | 9.27 | 6.63 | 0.33 |
|  | S | 16 | 1.53 | 2.26 | 1.36 | 0.09 |
|  | Ti | 22 | 1.10 | 1.62 | 0.65 | 0.08 |
|  | Fe | 26 | 0.91 | 1.35 | 0.47 | 0.08 |
|  | Na | 11 | 0.80 | 1.18 | 0.99 | 0.09 |
|  | Ca | 20 | 0.44 | 0.65 | 0.31 | 0.05 |
|  | Total | | 67.7 | 100 | 100 |  |
| **Spot4** | O | 8 | 25.67 | 32.19 | 37.75 | 3.89 |
|  | Si | 14 | 22.54 | 28.26 | 18.88 | 0.99 |
|  | Mg | 12 | 14.56 | 18.25 | 14.09 | 0.82 |
|  | C | 6 | 12.75 | 15.98 | 24.97 | 2.95 |
|  | Al | 13 | 2.09 | 2.63 | 1.83 | 0.15 |
|  | F | 9 | 1.31 | 1.65 | 1.63 | 0.46 |
|  | Na | 11 | 0.83 | 1.05 | 0.85 | 0.1 |
|  | Total | | 79.75 | 100 | 100 |  |
| **Spot5** | Ca | 20 | 35.39 | 65.96 | 42.31 | 1.1 |
|  | O | 8 | 12.54 | 23.38 | 37.56 | 2.35 |
|  | C | 6 | 4.34 | 8.08 | 17.29 | 0.85 |
|  | Mg | 12 | 0.81 | 1.51 | 1.59 | 0.09 |
|  | Al | 13 | 0.29 | 0.54 | 0.51 | 0.05 |
|  | F | 9 | 0.29 | 0.54 | 0.73 | 0.18 |
|  | Total | | 53.65 | 100 | 100 |  |
